# Development of Co-Rich Microwires with Graded Magnetic Anisotropy

**DOI:** 10.3390/s22010187

**Published:** 2021-12-28

**Authors:** Valentina Zhukova, Paula Corte-Leon, Juan Maria Blanco, Mihail Ipatov, Alvaro Gonzalez, Arcady Zhukov

**Affiliations:** 1 Departament of Advanced Polymers and Materials: Physics, Chemistry and Technology, Faculty of Chemistry, University of Basque Country, UPV/EHU, 20018 San Sebastian, Spain; paula.corte@ehu.eus (P.C.-L.); mihail.ipatov@ehu.es (M.I.); alvaro.gonzalezv@ehu.eus (A.G.); 2 Departamento de Física Aplicada, EIG, University of Basque Country, UPV/EHU, 20018 San Sebastian, Spain; juanmaria.blanco@ehu.es; 3 Ikerbasque, Basque Foundation for Science, 48011 Bilbao, Spain

**Keywords:** magnetic microwires, magnetic anisotropy, thermal treatment

## Abstract

In this paper, a gradual change in the hysteresis loop of Co-rich glass-coated microwire stress-annealed at variable temperature is observed. Such microwires annealed with a temperature gradient also present a variable squareness ratio and magnetic anisotropy field along the microwire’s length. The obtained graded anisotropy has been attributed to a gradual modification of the domain structure along the microwire originated by a counterbalance between shape, magnetoelastic, and induced magnetic anisotropies. Accordingly, we propose a rather simple route to design graded magnetic anisotropy in a magnetic microwire.

## 1. Introduction

Soft magnetic materials are highly demanded in many industries, such as automotive, aerospace and aviation, medicine, microelectronics, electrical engineering, magnetic refrigeration, home entertainment, energy conversion and harvesting, computer science, magnetic recording, sensorics, or electronic surveillance [1,2,3,4,5,6,7,8]. Although magnetic softness is of primary interest for such applications, the combination of mechanical properties (e.g., ductility, high tensile yield, elastic moduli, hardness), corrosion resistance, and downsizing are other important characteristics for industrial applications [9,10,11,12].

One of the general issues of traditional soft magnetic materials (e.g., Permalloy, Fe-Si, ferrites) is that their magnetic softness is substantially affected by processing. Accordingly, in most crystalline soft magnetic materials, a specially designed sequence of thermal treatments (including hot and cold rolling, annealing at carefully selected conditions (atmosphere, temperature), recrystallization) is needed to achieve good magnetic softness [1].

One of the most effective solutions is the use of amorphous soft magnetic materials with a liquid-like structure, prepared by rapid melt-quenching [6,8]. The magnetic softness of amorphous materials is usually associated with the absence of defects characteristic of crystalline magnets. Additionally, amorphous materials usually present better mechanical and corrosion properties [9,10,11,12,13]. Consequently, amorphous soft magnetic materials have been proposed for numerous applications, such as magnetic and magnetoelastic sensors, magnetic tags, smart composites, medicine, and so forth [4,6,7,8,14,15,16,17,18,19,20].

In the absence of magnetocrystalline anisotropy, shape anisotropy, and magnetoelastic anisotropy play a decisive role in the formation of the magnetic properties of amorphous materials. The best magnetic softness of amorphous materials can be generally achieved in nearly-zero magnetostrictive compositions. In amorphous alloys, the magnetostriction coefficient, *λ_s_*, is determined by their chemical composition [1,2,3,4,5,6]. Fe-rich amorphous alloys generally present positive and large *λ_s_* (about 40 × 10^−6^), while negative *λ_s_* (about −6 × 10^−6^) is commonly reported for Co-rich amorphous materials [21,22,23]. Accordingly, nearly-zero *λ_s_* values have been achieved in Co_x_Fe_1−x_ or Co_x_Mn_1−x_ (0 ≤ x ≤1) for 0.03 ≤ x ≤ 0.08 [21,22,23,24].

Both the magnetostriction coefficient, *λ_s_*, as well as the internal stresses, *σ_i_*, values are linked to the magnetoelastic anisotropy, *K_me_*, given as [4,18]:

*K_me_* ≈ 3/2 *λ_s_σ_i_*. (1)

Therefore, magnetic properties of amorphous materials are affected by the fabrication conditions (like quenching rate or internal stresses value and distribution) as well as by the chemical composition of the metallic alloy [18,22,23,24].

Additionally, the magnetic softness of amorphous materials is affected by a number of factors, like surface irregularities, intrinsic fluctuations of exchange energies and local anisotropies, clusters and chemical short-ordered regions, and relaxation effects due to local structural rearrangements linked to the fabrication process and post-processing [25,26].

Generally, amorphous materials obtained using rapidly quenching technology can be prepared in the shape of ribbons (planar geometry) and wires (cylindrical geometry) [21,22,23,24,25].

In soft magnetic materials with cylindrical symmetry and thin dimensions, the magnetization can be oriented along the long wire axis. Therefore, amorphous wires can present peculiar magnetic properties, like so-called magnetic bistability characterized by perfectly rectangular hysteresis loops [27,28,29,30].

Another interesting phenomenon, intrinsically related to magnetic softness, is the giant magnetoimpedance (GMI) effect, consisting of a substantial dependence of the impedance on the applied magnetic field [31,32,33,34,35]. The origin of the GMI effect is commonly attributed to the magnetic field dependence of skin depth, *δ*, of a magnetically soft conductor in terms of electrodynamics [31,32,33,34,35]. High circumferential magnetic permeability, typical for amorphous wires with nearly-zero *λ_s_*, is one of the pre-requisites for GMI effect optimization [30,31,32,33,34,35]. A high GMI ratio of amorphous wires (up to 650%) is proposed for various sensor applications [8,15].

Such versatile magnetic properties of amorphous wires stimulate extensive research related to the development of new technologies for amorphous wire preparation and on the optimization of magnetic softness in amorphous and nanocrystalline wires [7,8,16,17,18,19,20,21,23,24,25,26,27,28,29,30,31,32,33,35,36,37].

The so-called Taylor–Ulitovsky technique allows for magnetic wires of the widest diameter range to be manufactured (from 200 nm up to 100 μm), coated with an insulating and flexible glass coating [36,37,38].

An important advantage of the Taylor–Ulitovsky technology is that the wire diameter can be significantly reduced (usually by an order of magnitude). This diameter reduction is especially important for applications of magnetically bistable wires, since perfectly rectangular hysteresis loops can be observed only for wires with a minimum length [27,39].

Another advantage of glass-coated microwires is better corrosion resistance and (in some cases) mechanical properties provided by flexible, thin, biocompatible, and insulating glass coating. Such peculiarities of glass-coated microwires make them suitable for completely new applications, including biomedicine, non-destructive control of external stimuli (stress, temperature) in smart composites with microwire inclusions, magnetic memories and logics, magnetic and magnetoelastic sensors, or electronic surveillance [4,7,18,19,20,40,41,42].

It is worth mentioning that magnetic properties of glass-coated microwires can be tuned to meet the demands of a broad range of applications [42,43,44,45]. Several methods of tailoring magnetic properties have been reported, such as conventional furnace annealing, Joule heating, stress, and magnetic field annealing. Thermal treatment conditions (annealing temperature, *T_ann_*, time, *t_ann_*, stress, *σ*, current intensity, *I*) affect magnetic properties, such as coercivity, *H_c_*, the squareness ratio, *M_r_/M_o_*, GMI ratio, magnetic anisotropy field, *H_k_*, and so forth [42,43,44,45,46,47,48,49].

Recently, substantial increases in *H_c_* and *M_r_/M_o_* have been reported upon conventional furnace annealing of Co-rich microwires, while magnetic softness (decrease in *H_c_*, *M_r_/M_o_*, GMI ratio, *H_k_*) can be significantly improved by stress annealing [47,48,49].

While in the case of Co-rich stress-annealed microwires, the study of magnetic anisotropy has not been fully explored, in the case of Co-rich amorphous ribbons, stress-annealing-induced magnetic anisotropy and its influence on the off-diagonal GMI effect has already been reported and used for the construction of GMI sensors [50,51].

On the other hand, stress-annealing-induced magnetic anisotropy has been reported for Fe-rich microwires with positive *λ_s_* [44,52]. Additionally, the dependence of stress-annealing-induced magnetic anisotropy in Fe-rich microwires on annealing temperature has been proposed for the development of graded magnetic anisotropy [53]. The said graded magnetic anisotropy is obtained by a rather simple method consisting of annealing at fixed applied stress and a variable annealing temperature. Previously, graded magnetic anisotropy was obtained by a rather sophisticated method involving modification of the chemical composition during the sample preparation [54,55]. Magnetic materials with graded magnetic anisotropy presenting controllable spatial distribution of the magnetic anisotropy can show unusual magnetic properties: it was predicted [56] and recently experimentally shown [53,57] that materials with graded magnetic anisotropy can present controllable nucleation or pinning of domain walls.

The common features of Fe- and Co-rich microwires is that stress-annealing-induced anisotropy in both materials (Fe- and Co-rich microwires) is affected by several parameters, like *T_ann_*, *t_ann_*, *σ* [47,48,49,50,51,52,53]. Recently, it was observed that such stress-annealing in Fe-rich microwires is only partially reversible: subsequent annealing can recover only part of the stress-annealing-induced anisotropy [58,59]. Additionally, the said induced magnetic anisotropy is proportional to the *T_ann_*, *t_ann_*, *σ*—that is, the induced anisotropy increases when the *T_ann_* is increased under constant *t_ann_* and/or *σ* [58]. Accordingly, the parameter that can be modified during stress-annealing in the easiest way is *T_ann_.* Therefore, stress-annealing in a *T_ann_* gradient was proposed for the development of Fe-rich microwires with graded magnetic anisotropy [53,57].

Considering that, similarly to Fe-rich microwires, stress-annealing-induced magnetic anisotropy of Co-rich microwires is affected by *T_ann_*, *t_ann_*, *σ*, we intend to extend our studies to Co-rich microwires with the aim of developing Co-rich microwires with graded magnetic anisotropy.

Accordingly, in this paper, we present our recent experimental results on the preparation and processing of Co-rich magnetic microwires with graded magnetic anisotropy. Such graded magnetic anisotropy has been obtained by stress-annealing of Co-rich microwires at variable annealing temperatures.

## 2. Materials and Methods

Studied Co-rich glass-coated microwires have been prepared using the Taylor–Ulitovsky technique involving rapid melt quenching of metallic alloys covered by an insulating glass shell. The details of the preparation technique are described elsewhere [35,36,37]. We studied Co_64.04_Fe_5.71_B_15.88_Si_10.94_Cr_3.4_Ni_0.03_ (Sample 1) and Co_66_Cr_3.5_Fe_3.5_B_16_Si_11_ (Sample 2) glass-coated microwires with metallic nucleus diameters, *d*, of about 95 μm and 20.1 μm and a total diameter, *D*, of about 130 μm and 24.8 μm, respectively.

**Figure 1:**
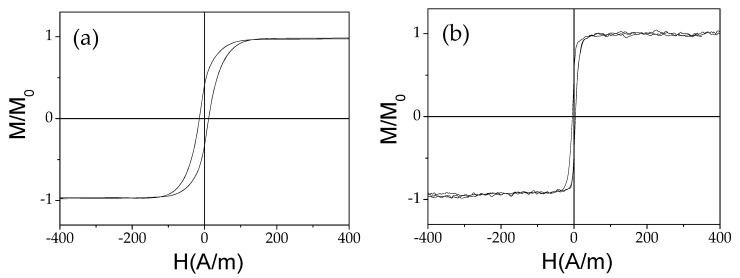
Hysteresis loops of as-prepared Co_64.04_Fe_5.71_B_15.88_Si_10.94_Cr_3.4_Ni_0.03_ (**a**) and Co_66_Cr_3.5_Fe_3.5_B_16_Si_11_ (**b**) microwires.

Both microwires present excellent magnetic softness with coercivities, *H_c_*, 14, and 3 A/m and magnetic anisotropy fields, *H_k_*, 100, and 40 A/m, respectively, typical for amorphous materials (see Figure 1).

Hysteresis loops in as-prepared and annealed samples were measured using the fluxmetric method detailed elsewhere [48]. For a better comparison of samples with different composition and geometry and subjected to different heat treatments, the hysteresis loops are presented as the dependence of the normalized magnetization, *M/M*_0_, (where *M* is the magnetization in a given magnetic field, and *M*_0_ is the magnetization in the maximum applied field) on the magnetic field, *H.* The magnetic field is created by a long (about 12 cm long) and thin (about 8 mm in diameter) solenoid. As described elsewhere, the magnetic field generated by such a solenoid is fairly uniform along the axis over a sufficiently long (about 90% of the solenoid length) region [60,61].

The hysteresis loops of samples as-prepared and stress-annealed at a constant temperature were measured using a pick-up coil that was 20 mm long. In this case, the sample length was 50 mm, and each sample was placed inside the solenoid which was 120 mm long. The variation of the hysteresis loops along the sample length was evaluated using a short (2 mm long), movable pick-up coil, as also recently reported for Fe-rich microwires [57]. In both cases, it is essentially relevant that the pick-up coil lengths (2 mm and 20 mm, respectively) are much shorter than the length of the magnetizing coil (120 mm). Therefore, the sample is magnetized by a rather uniform magnetic field at the sample portion where the magnetization is measured by the pick-up coils.

The magnetostriction coefficient, *λ_s_*, determined by the small angle magnetization rotation (SAMR) method adapted for glass-coated microwires [23] gives values of *λ_s_* ≈ −1 × 10^−7^ (sample 1) and −0.3 × 10^−7^ (sample 2), respectively.

It is worth mentioning that, usually, the Taylor–Ulitovsky technique is employed for preparation of thinner microwires (typically with diameters, *d*, of a metallic nucleus below 50 µm). However, recent preparation of Fe-rich glass-coated microwires with *d* ≥ 100 μm was reported [38]. Such “thick” microwires present certain interest for industrial applications, like non-contact stress monitoring or magnetic tags [18,40]. The magnetic properties of “thick” Fe-rich glass-coated microwires are rather different from those of thinner Fe-rich glass-coated microwires [38]. Therefore, for the present studies, we have selected two Co-rich microwires with fairly similar compositions, but with rather different *d*-values.

**Figure 2:**
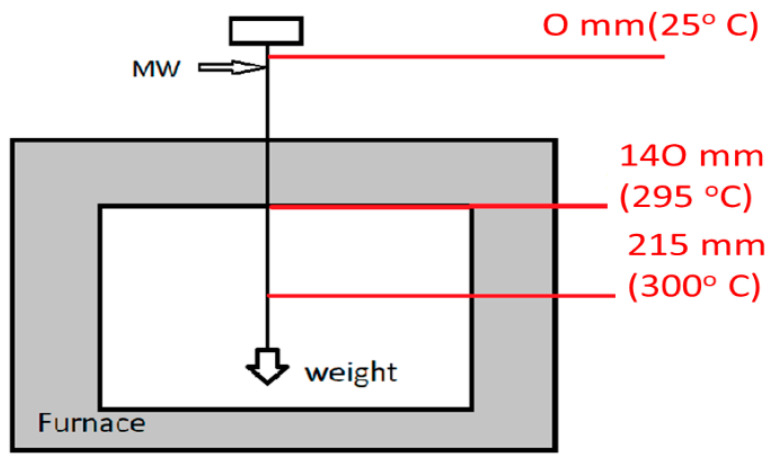
Scheme of stress-annealing at variable temperatures.

Both samples were annealed in a standard Thermolyne furnace. The annealing at variable temperature was performed in a similar way as for Fe-rich microwires [57]: a 30 cm long microwire was annealed in a conventional furnace under tensile stress, *σ,* created by a mechanical load, *P*, attached to one end of the microwire (see Figure 2). Such *σ*—magnitude was obtained considering the composite structure of microwires consisting of a metallic nucleus and glass coating with different Young moduli (*E*_2_—for the metal and *E*_1_—for the glass coating), as previously described elsewhere [49,50,51,52,53,58]:

(2) $$\sigma =\frac{K\cdot P}{K\cdot S_{m}+S_{gl}}$$

being *K = E*_2_*/E*_1_ and *S_m_* and *S_gl_* being the metallic nucleus and glass coating cross-sections, respectively.

In previous publications [49,50,51,62], Young moduli at room temperature for pure metal (*E*_2_ = 1.5 × 10^11^ Pa) and Pyrex glass (*E*_1_ = 7.5 × 10^10^ Pa) were used for stresses estimation. Accordingly, the coefficient *K* = *E*_2_*/E*_1_ ≈ 2 was used.

In fact, for a more correct estimation of *E*_2_ and *E*_1_ values during stress-annealing, it is necessary to consider *E*_2_ and *E*_1_ values at the annealing temperature. Additionally, it is more correct to consider *E*_2_ values for amorphous alloys (not for pure metals).

A decrease in both *E*_2_ and *E*_1_ upon heating was experimentally observed [63,64]. From the experimentally measured *E*_2_ and *E*_1_ temperature dependencies [63,64], the following *E*-values can be considered: *E*_2_ = 14,500 kg/cm^2^ [63] and *E*_1_ = 7400 kg/cm^2^ [64]. Although these *E*_2_ and *E*_1_-values are different from those previously considered [49,50,51,60], the *K*-coefficient given as *E*_2_*/E*_1_ remains the same: *K* = *E*_2_*/E*_1_ ≈ 2. For the present case, *σ ≈* 500 MPa and 400 MPa were used for samples 1 and 2, respectively.

One end of the studied microwire was introduced into the furnace through the orifice up to the zone with constant temperature, *T*, while the opposite sample end remained outside the furnace. Like in any real furnace, there is a zone with constant *T*, in the middle of the furnace, while near the ends the temperature is more general, and hence, a temperature gradient exists near the ends of the furnace [65].

**Figure 3:**
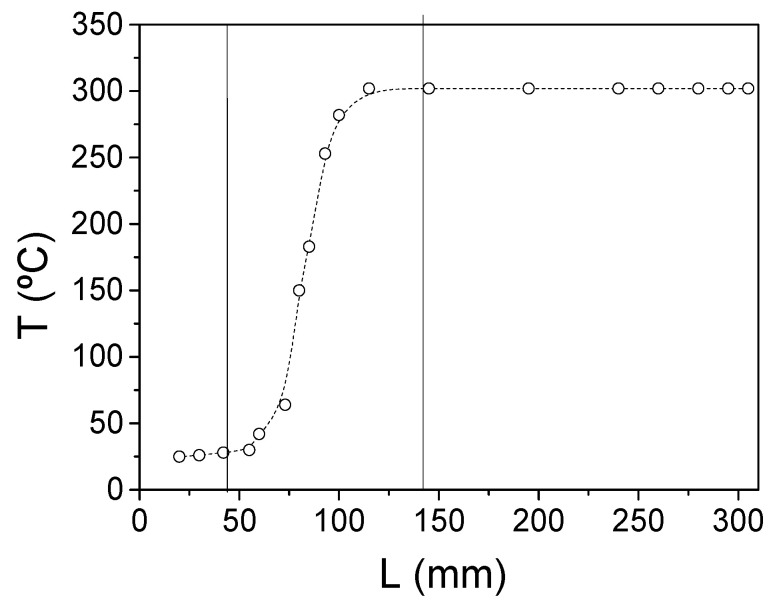
Temperature distribution inside the furnace for the temperature set to 300 °C.

The temperature distribution, measured using a commercial (NiCr-Ni) thermocouple (when *T* is set to 300 °C) inside the furnace, in the orifice (through which the microwire is introduced into the furnace) and near the furnace is shown in Figure 3. Consequently, part of the sample placed in the zone with constant *T* was stress-annealed at a fixed temperature and part of the sample placed in the temperature gradient zone was stress-annealed under variable *T*.

Finally, the microwire placed outside the furnace was just loaded and unloaded. The presence of an insulating glass-coating allows for annealing of microwires in air. Similar stress-annealing conditions (*T_ann_*, *σ*) were used in previous publications [47,48,49]. The integrity of microwires before and after stress-annealing was checked by optical microscopy.

One of the most important advantages of amorphous materials is better mechanical properties (e.g., high ductility). Therefore, we chose an annealing temperature, *T_ann_*, (300 °C) well below the crystallization temperature. In previous publications, the most significant change in magnetic anisotropy for selected *T_ann_* in Co-rich microwires was observed at *σ* ≈ 350 MPa [47,48,49]. Correspondingly, the mechanical load was selected, allowing to apply *σ* ≈ 400 and 500 MPa.

**Figure 4:**
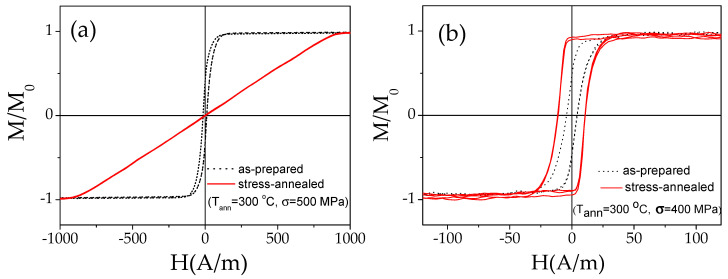
Effect of stress-annealing on hysteresis loops of sample 1 (**a**) and sample 2 (**b**).

The crystallization, *T_cr_*, and Curie, *T_c_*, temperatures of the studied microwires, evaluated by the DSC method as described elsewhere [66], were about 510 and 370 °C, respectively. *T_cr_* and *T_c_*, values are consistent with the values observed in CoFeCrSiB microwires [46] and ribbons [67,68] with similar compositions. Consequently, like other Co-rich stress-annealed microwires, the studied microwires annealed at *T_ann_* ≤ 300 °C retain amorphous structure after stress-annealing [47,48,49]. The amorphous structure of stress-annealed microwires is also indirectly confirmed by the excellent magnetic softness (see Figure 4) and ductility typical for amorphous materials—substantial magnetic hardening has been reported elsewhere at the onset of crystallization [67].

## 3. Experimental Results and Discussion

- Dependence of hysteresis loops on stress-annealing conditions;
- Variation of magnetic properties along the wires associated with temperature gradients during stress-annealing.

A modification of the hysteresis loops of studied samples was observed upon stress- annealing (see Figure 4). As mentioned above, the hysteresis loops of Co-rich microwires with vanishing *λ_s_*-values are usually substantially affected by stress-annealing [42,47,48].

Thus, an almost anhysteretic loop with *H_k_* ≈ 900 A/m is observed in sample 1 stress-annealed at *T_ann_* = 300 °C (see Figure 4a). Such a character of the hysteresis loop must be attributed to a transverse character of stress-annealing-induced magnetic anisotropy and magnetization processes by magnetization rotation. The opposite tendency is observed for sample 2: the hysteresis loop becomes almost perfectly rectangular with *H_c_* ≈ 11 A/m (see Figure 4b).

In these experiments, we used the same *T_ann_*, and *t_ann_*, while *σ*–magnitudes evaluated from Equation (2) were 500 and 400 MPa. Therefore, the observed difference in the effect of stress-annealing on hysteresis loops of studied samples (see Figure 4a,b) can be attributed to different *σ*–magnitudes. As recently reported [53], even in the same Co-rich microwire, the stress-annealing-induced anisotropy is considerably affected by the *σ*-values: in Fe_3.6_Co_69.2_Ni_1_B_12.5_Si_11_Mo_1.5_C_1.2_ microwires for *σ* ≥ 470 MPa, the character of the induced anisotropy changed from axial to transverse [53]. Alternatively, *λ_s_* is affected by stresses (either applied or internal) [69]. Such stress-dependence of *λ_s_* can be essentially relevant for Co-rich amorphous materials with vanishing *λ_s_*. Therefore, annealing can affect not only the *λ_s_*-value, but even its sign [52]. Accordingly, observed differences of hysteresis loops in both stress-annealed samples can also be related to different chemical compositions, and hence to *λ_s_*-values.

As recently shown, the stress-annealing-induced magnetic anisotropy of microwires depends on *T_ann_*, *t_ann_*, and *σ* [53,57,70]. Accordingly, stress-annealing of Fe-rich microwires in the *T_ann_* gradient was employed for realization of controllable spatial distribution of the magnetic anisotropy, that is, for development of graded magnetic anisotropy [53,57].

Below, we use the same concept for studied Co-rich microwires. Similarly to previous publications on graded anisotropy in Fe-rich microwires, we used the dependence of the stress-annealing-induced anisotropy on the annealing temperature, *T_ann_* [53,57,70].

**Figure 5:**
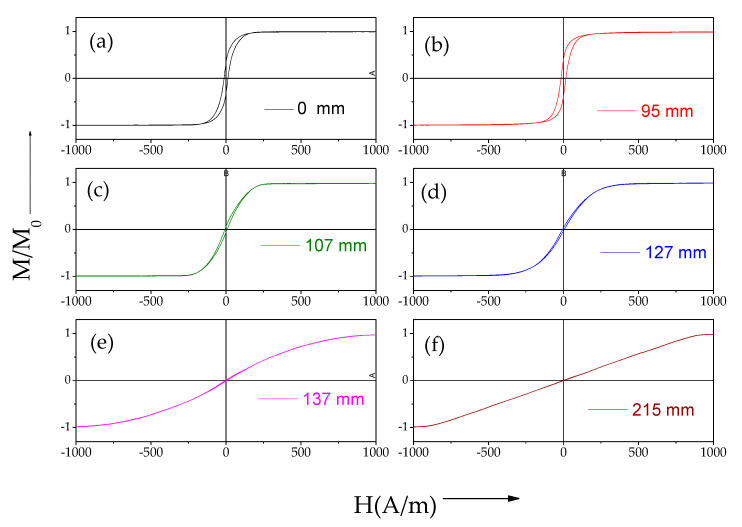
Hysteresis loops of sample 1 stress-annealed variable *T_ann_* and *σ* ≈ 500 MPa. *T_ann_* evaluated from Figure 3 are: 25 °C (**a**); 249 °C (**b**); 277 °C (**c**); 290 °C (**d**); 295 °C (**e**); and 300 °C (**f**), respectively.

**Figure 6:**
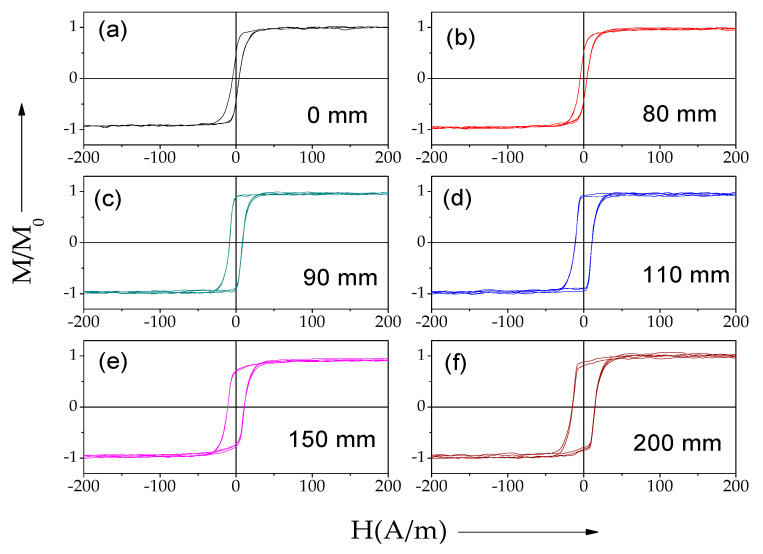
Hysteresis loops of sample 2 stress-annealed variable *T_ann_* and *σ ≈* 400 MPa. *T_ann_* evaluated from Figure 3 are: 25 °C (**a**); 160 °C (**b**); 251 °C (**c**); 290 °C (**d**); 295 °C (**e**); and 300 °C (**f**), respectively.

As shown in Figure 5 and Figure 6, a gradual change in the hysteresis loop (measured by the short pick-up coil) of both samples stress-annealed at variable *T_ann_* is observed.

**Figure 7:**
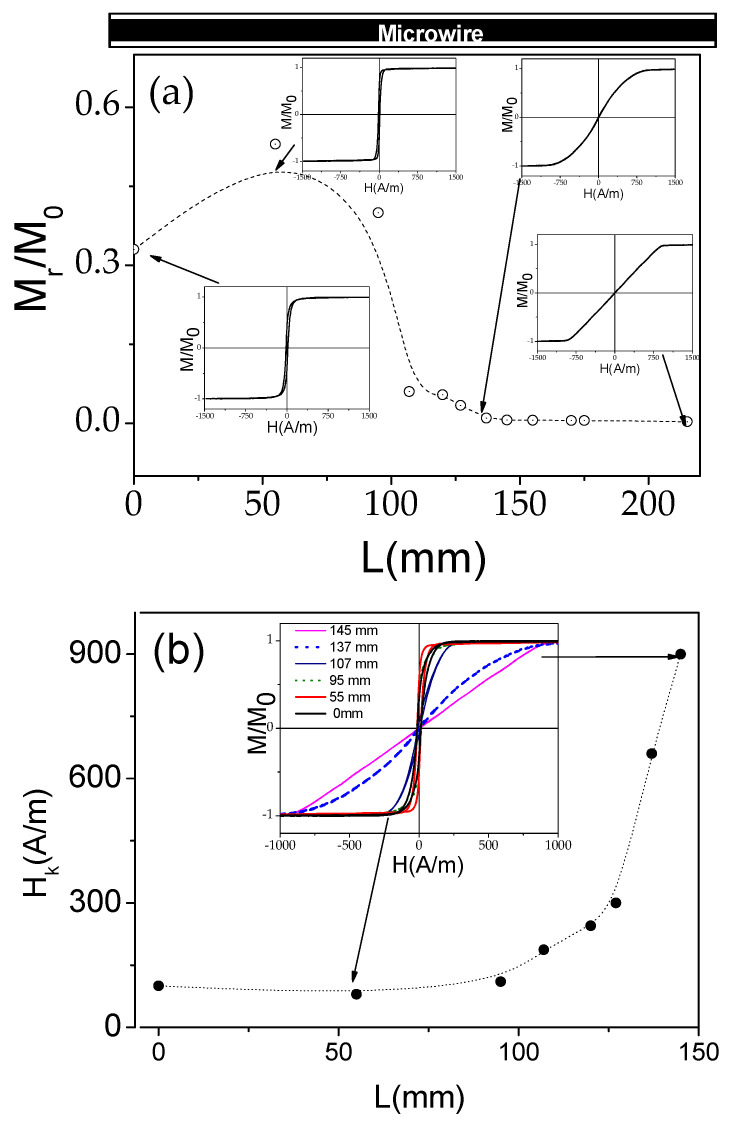
Variation of *M_r_/M*_0_ (**a**) and *H_k_* (**b**) in sample 1 annealed at variable *T_ann_*. The lines are just guides for the eyes.

Both samples present variation of their magnetic properties along their lengths associated with the *T_ann_* gradient produced during stress-annealing (see Figure 5 and Figure 6). Thus, we can deduce that sample 1 presents variable remanent magnetization, *M_r_/M*_0_, and a magnetic anisotropy field, *H_k_*, along the microwire length, *L* (see Figure 7a,b).

**Figure 8:**
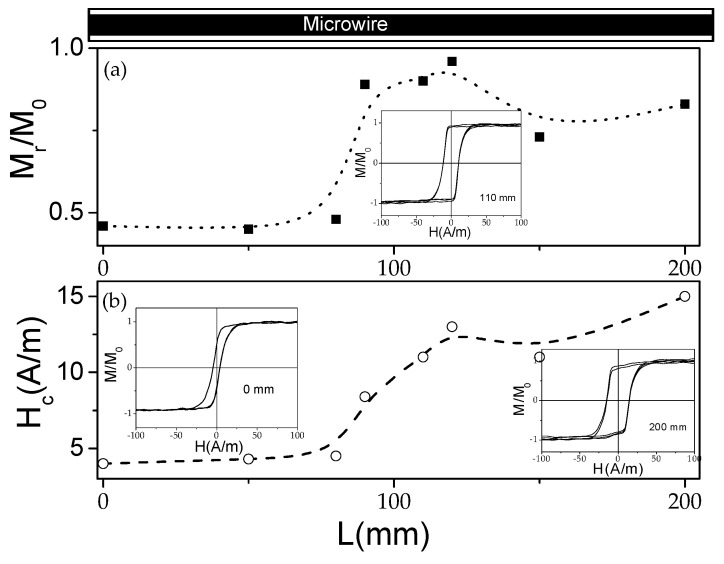
Variation of *M_r_/M*_0_ (**a**) and *H_c_* (**b**) in the sample 2 annealed at variable *T_ann_*. The lines are just guides for the eyes.

Similarly, a modification in the remanent magnetization, *M_r_/M*_0_, and the coercivity, *H_c_*, is observed in sample 2 subjected to stress-annealing in the *T_ann_* gradient (see Figure 8).

Generally, the evolution of magnetic properties upon stress-annealing in *T_ann_* gradients observed in both samples presents features similar to the ones already reported for stress-annealing-induced magnetic anisotropy in Co-rich microwires [47,48,49]. Thus, from a comparison of Figure 3, Figure 7a and Figure 8a, we can deduce an increase in *M_r_/M*_0_ followed by a decrease with increasing *T_ann_* in both samples. Additionally, almost anhysteretic loops with high enough *H_k_,* observed in the sample 1 portion subjected to stress-annealing at sufficiently high *T_ann_* must be attributed to transverse magnetic anisotropy and remagnetization by magnetization rotation.

**Figure 9:**
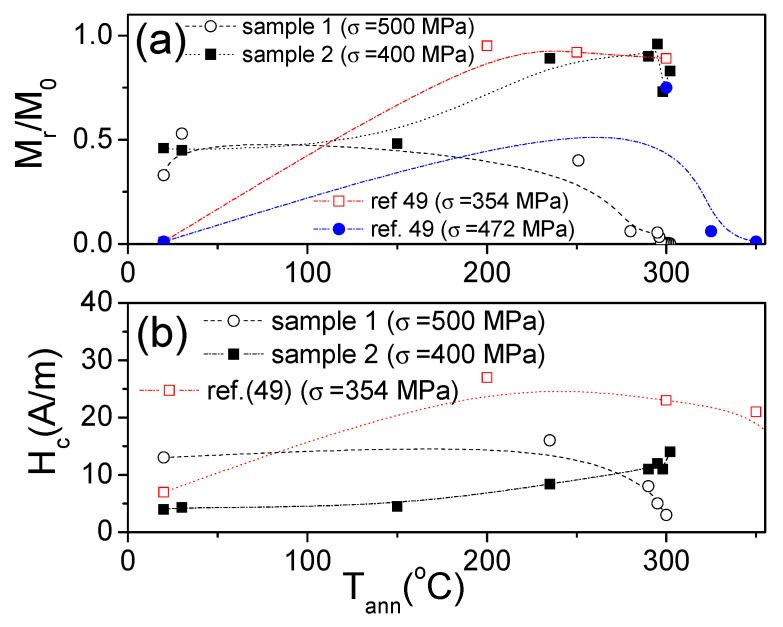
Comparison of *M_r_/M*_0_ *(T_ann_)* (**a**) and *H_c_ (T_ann_)* (**b**) dependencies for samples 1 and 2 and for the Co_69.2_Fe_3.6_Ni_1_B_12.5_Si_11_Mo_1.5_C_1.2_ microwires studied in the Refs. [47,48,49]. The lines are just guides for the eyes.

An attempt to compare *M_r_/M*_0_(*T_ann_*) and *H_c_*(*T_ann_*) dependencies evaluated from Figure 5, Figure 6, Figure 7 and Figure 8 and similar dependencies previously reported for the Co_69.2_Fe_3.6_Ni_1_B_12.5_Si_11_Mo_1.5_C_1.2_ microwire is provided in Figure 9.

It should be noted that the experimental data were obtained in different conditions:

(i) Co_69.2_Fe_3.6_Ni_1_B_12.5_Si_11_Mo_1.5_C_1.2_ microwires studied in the Refs. [47,48,49] were annealed each time at different temperatures, with the sample being placed in the furnace zone with stable *T*. On the other hand, samples 1 and 2 were placed into the furnace zone with variable *T*, and therefore a *T_ann_* spectrum from room temperature to 300 °C can be realized in the same sample.
(ii) Although all Co-rich microwires present vanishing *λ_s_*-values, even as-prepared samples have different hysteresis loops. Such difference can be attributed to slightly different chemical compositions and different geometries.
(iii) Additionally, the stress, *σ*, applied during annealing under mechanical load, evaluated by Equation (2), is different for all studied microwires due to different *d*- and *D*-values.

Accordingly, the as-prepared Co_69.2_Fe_3.6_Ni_1_B_12.5_Si_11_Mo_1.5_C_1.2_ microwire studied in refs. [47,48,49] presents lower *M_r_/M*_0_ and *H_c_*-values than samples 1 and 2. However, despite the aforementioned differences, there are a few common features. Thus, an increase in *M_r_/M_o_* versus *T_ann_* is observed in the Co_69.2_Fe_3.6_Ni_1_B_12.5_Si_11_Mo_1.5_C_1.2_ microwire, annealed at *σ* ≈ 354 MPa, and in sample 2, annealed at *σ* ≈ 400 MPa (see Figure 9a). However, the *M_r_/M_o_*(*T_ann_*) dependence for a Co_69.2_Fe_3.6_Ni_1_B_12.5_Si_11_Mo_1.5_C_1.2_ microwire annealed at *σ* ≈ 472 MPa shows a slight increase followed by a decrease in *M_r_/M_o_*. Similarly, a decrease in *M_r_/M_o_* versus *T_ann_* is observed for sample 1 annealed at *σ* ≈ 500 MPa (see Figure 9a). From the provided comparison, it can be deduced that there is a general tendency toward a decrease in *M_r_/M_o_* upon annealing at sufficiently high *T_ann_* and *σ*.

Some tendency can also be extracted from the *H_c_*(*T_ann_*) dependencies. Thus, for a Co_69.2_Fe_3.6_Ni_1_B_12.5_Si_11_Mo_1.5_C_1.2_ microwire annealed at *σ* ≈ 354 MPa, an increase in *H_c_*(*T_ann_*) is observed, followed by a decrease (Figure 9b), while a decrease in *H_c_* is observed in sample 1 annealed at *σ* ≈ 500 MPa (see Figure 9b).

On the other hand, saturation magnetization of both samples remains almost unchanged: magnetic moment values evaluated by measurements at *H* = 1T (using VSM magnetometer) were almost the same. Such features of stress-annealed samples must be attributed to the amorphous structure of as-prepared and all stress-annealed samples. Indeed, the influence of annealing on saturation magnetization can be relevant upon devitrification of the amorphous precursor [71]. However, in amorphous materials, the saturation magnetization value is determined by the chemical composition [22]. It is generally accepted and even experimentally proved that the saturation magnetization of amorphous materials is almost independent of annealing [72].

Differences between the two studied samples must be attributed to (i) different *λ_s_*-values (as their chemical compositions are different) and (ii) different *σ*-values (500 MPa and 400 MPa, respectively).

The modification of the hysteresis loops of the studied microwires must be related to the magnetic domain structure of microwires, which is affected by the *λ_s_*-value and sign, the internal stresses distribution, and the shape magnetic anisotropy. Thus, the axial magnetization alignment promoted by the exchange energy contribution is especially relevant for the case of thin and long enough magnetic wires due to high shape anisotropy [27,73,74]. However, in Co-rich magnetic microwires with *λ_s_* < 0, the presence of internal stresses is another factor that affects magnetoelastic anisotropy, *K_me_* given by Equation (1).

The origin of internal stresses in glass-coated microwires has been discussed in several publications [62,75,76,77,78,79,80,81]. In addition to quenching internal stresses, *σ_iq_*, arising from the rapid melt quenching itself [80], there are two more internal stresses contributions: the difference in thermal expansion coefficients of metallic alloy and glass coating, *σ_it_*, and the drawing stresses, *σ_id_* [62,75,76,77,78,79,81]. Generally, various theoretical approaches and indirect experimental results (e.g., the effect of glass-coating etching, the influence of applied stresses) show that the *σ_it_* contribution is the most relevant [42,62,75,76,77,78,79]. Accordingly, *σ_it_* » *σ_iq_* and *σ_it_* » *σ_id_*. The most simplified, *σ_it_*, estimation gives [49,77,79]:

*σ_φ_* = *σ_r_* = *P* = *εEk*Δ/(*k*/3 + 1)Δ + 4/3; *σ_z_* = *P*(*k* + 1)Δ + 2/(*k*Δ + 1), (3)

where *σ_φ_*, *σ_r_*, and *σ_z_* are circular, radial, and axial internal stress components; Δ = (1 − *ρ*^2^)/*ρ*^2^, *ρ* is the geometric ratio, given as *d/D*; k =*E_g_/E_m_*, *E_m_, E_g_* Young moduli of a metallic nucleus and glass, respectively; *ε* = (*α_m_* − *α_g_)(T_m_* − *T_room_)*, *α_m_*, *α_g_* are thermal expansion coefficients of a metallic nucleus and glass, respectively; and *T_m_*, *T_room_* are melting and room temperatures.

Accordingly, *σ_it_* presents the tensor character and is affected by the microwire geometry through the *ρ*-ratio *(ρ = d/D)*. Additionally, in most parts of the metallic nucleus volume (roughly up to *r* ~0.85 *R*, where *R* is the metallic nucleus radius), the axial component, *σ_z_*, is approximately an order of magnitude higher than *σ_φ_* and *σ_r_* [75,76,77,78,79,80,81]. Such a character of internal stress turns the magnetization of Co-rich microwires with *λ_s_* < 0 to a circumferential direction [49,73,74].

Consequently, the counterbalance between the shape magnetic anisotropy, the magnetoelastic anisotropy, and the stress-annealing-induced anisotropy determines the hysteresis loops modification observed in Figure 7 and Figure 8.

In terms of the core-shell domain structure model, the modification in remanent magnetization, *M_r_/M_o_*, along the microwire can be attributed to the change in the inner axially magnetized core radius, *R_c_*, considering its relationship with *M_r_/M*_0_, given as [27,73,74]:

*R_c_* = *R*(*M_r_*/*M*_0_)^1/2^, (4)

where *R* is the metallic nucleus radius.

Accordingly, the observed spatial distribution of hysteresis loops must be attributed to a gradual modification of the domain structure along the length of the microwires stress-annealed in a temperature gradient.

Previously, the circumferential magnetization orientation of the outer domain shell in Co-rich microwires has been observed by the magneto-optical Kerr Effect (MOKE) method under applied stress [82] and deduced from magnetic field dependencies of the GMI ratio, Δ*Z/Z*, in stress-annealed Co-rich microwires with rectangular hysteresis loops [48,49,83]. Such circumferential magnetization orientations in stress-annealed Co-rich microwires have been evidenced by double-peak Δ*Z/Z(H)* dependencies, observed at high enough (above 100 MHz) frequencies [48,49,83]. Double-peak Δ*Z/Z(H)* dependencies have been predicted and experimentally reported for magnetic wires with circumferential magnetic anisotropy [31,32,33,34,84].

Coexistence of double-peak Δ*Z/Z*(*H*) dependencies and rectangular bulk hysteresis loops in stress-annealed Co-rich microwires has been explained considering the frequency dependence of the skin penetration depth, *δ,* as well as the existence of thin surface layers with circumferential magnetic anisotropy [48,49,83]. At relatively low frequencies, the skin depth is comparable and even higher than the microwire radius, and therefore, the current flows through the whole ferromagnetic nucleus. However, as the frequency rises, the current flows closer to the surface [83].

Therefore, the modification of a magnetic domain structure upon stress-annealing consists of a change in the volume of the outer domain shell with circumferential magnetization orientation at the expense of the inner axially magnetized core.

Previously, a much more sophisticated technology involving a change in the chemical composition during deposition was proposed to obtain thin films with graded magnetic anisotropy [54,55]. Magnetic materials with graded properties have been proposed for various applications, including magnetic recording, manipulations of electromagnetic waves, or controllable domain wall propagation [54,55,56,57,85,86]. Thus, recently, controllable domain wall-breaking or -trapping have been reported for Fe-rich magnetic microwires with graded magnetic anisotropy [57].

The influence of various parameters, such as annealing temperature, applied stress during annealing, or annealing time on the magnetic anisotropy of Co-rich microwires with vanishing magnetostriction was recently demonstrated by us in several publications [49,50,51,82]. The only parameter that can be easily changed during the same experiment is the annealing temperature. We previously used this solution for the preparation of Fe-rich microwires with graded magnetic anisotropy. Accordingly, we selected Co-rich microwires of two different compositions and geometries to prove that, as with Fe-rich microwires, such processing can be used to obtain Co-rich microwires with graded magnetic anisotropy.

Obtained Co-rich microwires with gradual sensibility to magnetic fields are suitable for the development of magnetic sensors capable of detecting both a magnetic field and its approximate location along the microwire length. For example, the dependence of the sample magnetization measured in a given magnetic field versus their position along the sample may be suitable for position sensors. Additionally, the studied samples have been shown to exhibit excellent magnetic softness, and hence can be good candidates for GMI-related technologies, as magnetic softness has been reported to be related to high GMI effectiveness. On the other hand, thick Co-rich microwires with tunable harmonics can be useful for various applications, such as security and electronic surveillance or smart composites for non-destructive control applications. Stress-annealing-induced magnetic anisotropy is a useful tool for improvement of the stress sensitivity of the magnetic properties of magnetic microwires [87]. Therefore, the use of Co-rich microwires with graded magnetic anisotropy is expected to improve the performance of stress-sensitive composites with microwire inclusions.

Accordingly, we propose a rather simple route involving stress-annealing in a temperature gradient to design graded magnetic anisotropy in Co-rich magnetic microwires.

## 4. Conclusions

In this paper, we proposed a rather simple method for preparation of Co-rich microwires with graded magnetic anisotropy consisting of stress-annealing under a temperature gradient. A gradual change in the hysteresis loop of a Co-rich glass-coated microwire stress-annealed at variable temperature was observed. Additionally, such microwires stress-annealed with a temperature gradient also presented with a variable squareness ratio and magnetic anisotropy field along the microwire length. Obtained graded anisotropy has been attributed to a gradual modification of the domain structure along the microwire originated by a counterbalance between shape, magnetoelastic, and induced magnetic anisotropies. The gradual sensibility to magnetic fields would allow for the development of magnetic sensors capable of detecting both a magnetic field and its approximate location along the microwire length. Studied samples have shown to exhibit excellent magnetic softness, and hence can be a good candidate for GMI-related technologies, as magnetic softness has been reported to be related to high GMI effectiveness. On the other hand, thick microwires with tunable harmonics can be useful for security and electronic surveillance and for non-destructive control applications.

## Data Availability

Data available on request due to restrictions related to the developing projects.
